# Chronic Diclofenac Exposure Increases Mitochondrial Oxidative Stress, Inflammatory Mediators, and Cardiac Dysfunction

**DOI:** 10.1007/s10557-021-07253-4

**Published:** 2021-09-09

**Authors:** Phung N. Thai, Lu Ren, Wilson Xu, James Overton, Valeriy Timofeyev, Carol E. Nader, Michael Haddad, Jun Yang, Aldrin V Gomes, Bruce D. Hammock, Nipavan Chiamvimonvat, Padmini Sirish

**Affiliations:** 1 Department of Internal Medicine, Division of Cardiovascular Medicine, University of California, Davis, 451 Health Science Drive, CA 95616 Davis, USA; 2 Department of Entomology and Nematology and Comprehensive Cancer Center, University of California, Davis, CA USA; 3 Department of Physiology and Membrane Biology, University of California, Davis, CA USA; 4 Department of Pharmacology, University of California, Davis, CA USA; 5 Department of Veterans Affairs, Northern California Health Care System, 10535 Hospital Way, Mather, CA 95655 USA

**Keywords:** Mitochondria, Oxidative stress, Nonsteroidal anti-inflammatory drug, Diclofenac

## Abstract

**Purpose:**

Nonsteroidal anti-inflammatory drugs (NSAIDs) are among one of the most commonly prescribed medications for pain and inflammation. Diclofenac (DIC) is a commonly prescribed NSAID that is known to increase the risk of cardiovascular diseases. However, the mechanisms underlying its cardiotoxic effects remain largely unknown. In this study, we tested the hypothesis that chronic exposure to DIC increases oxidative stress, which ultimately impairs cardiovascular function.

**Methods and Results:**

Mice were treated with DIC for 4 weeks and subsequently subjected to in vivo and in vitro functional assessments. Chronic DIC exposure resulted in not only systolic but also diastolic dysfunction. DIC treatment, however, did not alter blood pressure or electrocardiographic recordings. Importantly, treatment with DIC significantly increased inflammatory cytokines and chemokines as well as cardiac fibroblast activation and proliferation. There was increased reactive oxygen species (ROS) production in cardiomyocytes from DIC-treated mice, which may contribute to the more depolarized mitochondrial membrane potential and reduced energy production, leading to a significant decrease in sarcoplasmic reticulum (SR) Ca^2+^ load, Ca^2+^ transients, and sarcomere shortening. Using unbiased metabolomic analyses, we demonstrated significant alterations in oxylipin profiles towards inflammatory features in chronic DIC treatment.

**Conclusions:**

Together, chronic treatment with DIC resulted in severe cardiotoxicity, which was mediated, in part, by an increase in mitochondrial oxidative stress.

**Supplementary Information:**

The online version contains supplementary material available at 10.1007/s10557-021-07253-4.

## Introduction

Nonsteroidal anti-inflammatory drugs (NSAIDs) are widely available over the counter and commonly prescribed in the USA [1]. Although they have a relatively good safety profile, their widespread availability, uncontrolled disposal, and improper usage can be detrimental to the environment and human health. Indeed, improper disposal of these drugs leads to their accumulation in the water system [2], that are harmful to human health [3]. Additionally, since NSAIDs are so common and exist in many varieties that are often prescribed with other drugs, it is easy to unknowingly exceed the recommended dosage [4]. One of the known adverse effects of taking NSAIDs is elevated risks for cardiovascular complications, such as elevated blood pressure and myocardial infarction [5]. However, the exact mechanism underlying the increased susceptibility to these cardiovascular events is not completely understood.

One of the critical signaling pathways activated during inflammation is the arachidonic acid (ARA) pathway. NSAIDs exert their inflammatory actions by targeting the enzymes cyclooxygenase (COX)-1 and COX-2 in the ARA metabolic pathway. Although NSAIDs predominantly induce their effects on these enzymes, they do so with varying degrees [6]. The actions of COX-1 and COX-2 on ARAs result in the production of prostaglandins and thromboxane including prostaglandin E_2_ (PGE_2_), prostaglandin D_2_ (PGD_2_), prostaglandin F_2α_ (PGF_2α_), prostacyclin (PGI_2_), thromboxane (TXA2), and related compounds that contribute to the inflammatory response [7]. Indeed, the differential level of inhibition as well as differential inhibition of COX-1 and COX-2 may produce varying toxicity [8]. Additionally, with the widespread availability of NSAIDs, their common usage, and improper disposal, the cumulative, inhibitory effects of the COX enzymes can result in profound cardiovascular consequences.

Recent emerging evidence has suggested that inhibition of the COX enzymes induces oxidative stress [9]. The mitochondria are the primary site for reactive oxygen species (ROS) production through their role in energy generation. This energy generation, although efficient, is prone to produce ROS, which normally are scavenged and detoxified by the cellular antioxidant system [10]. Under conditions of increased chronic oxidative stress, however, ROS generation can be overwhelming and detrimental to cellular function and may induce apoptosis [11]. Previous studies have demonstrated that ROS can directly increase the production of extracellular matrix as well as mitochondrial structural and membrane potential damage, leading to cardiac fibrosis [12]. Since the heart is an energetically demanding organ, it is comprised of a dense network of mitochondria. It is estimated that about 25–30% of the cardiomyocytes volume is occupied by mitochondria [13] and the heart is particularly susceptible to damage through oxidative stress, which can result in a plethora of cardiovascular complications including cardiac fibrosis and dysfunction [14]. It is well documented that the risk for cardiovascular events increases with the usage of NSAIDs [15]; however, it remains unclear what are the underlying mechanisms.

A recent in vitro study involving exposure of immortalized human cardiomyocytes to diclofenac (DIC) demonstrated an increase in ROS production, a decrease in mitochondrial membrane potential, and cardiotoxicity leading to cell death [16]. In our study, we tested the hypothesis that chronic treatment with a commonly prescribed NSAID, DIC, increases oxidative stress, which ultimately impairs cardiac function in vivo. Chronic treatment with DIC results in significant systolic and diastolic dysfunction as assessed by in vivo echocardiography with a marked decrease in single-cell shortening and Ca^2+^ transients. Metabolomic profiling, single-cell flow cytometric analyses, and mitochondrial function were performed to determine the underlying mechanisms of cardiac dysfunction.

## Materials and Methods

### Echocardiography

Eight to 12-week-old male and female C57BL/6 mice were treated with DIC (15 mg/kg/day) or vehicle alone in drinking water for 4 weeks based on our previous studies and subsequently monitored in accordance with approved protocols of the IACUC Committee at the University of California, Davis. Systolic function (M-mode images) was measured in conscious mice and diastolic function (pulse-wave Doppler and tissue Doppler) was measured in the presence of 0.5–1% isoflurane.

### Blood Pressure Recordings

Mice were acclimated to the non-invasive, tail vein blood pressure recording system (Kent Scientific Corporation) for 5 days before actual measurements were taken.

### Mitochondrial Membrane Potential

Mitochondrial membrane potential was assessed in freshly isolated control and DIC-treated cardiomyocytes using tetramethylrodamine ester (TMRM) [17]. Cardiomyocytes were incubated with TMRM and the recordings analyzed using ImageJ FIJI Software.

### Mitochondrial Ca^2+^ Uptake

Mitochondrial Ca^2+^ uptake was monitored in freshly isolated control and DIC-treated cardiomyocytes using X-Rhod-1 AM (ThermoFisher Scientific) [17]. Cells were loaded with X-Rhod-1 AM for 40 min at 37 °C. Mitochondrial Ca^2+^ uptake was monitored by the change in fluorescence intensity, normalized to the baseline fluorescence intensity, after the addition of 5 μM Ca^2+^ and 10 μM Ca^2+^.

###  ROS Production

Superoxide generation was monitored in freshly isolated control and DIC-treated cardiomyocytes using MitoSox Red (ThermoFisher Scientific) [17]. Intact cells were loaded with MitoSox Red for 30 min at 37 °C. ROS generation was monitored after field stimulation (0.5 Hz and 4 Hz) and application of 100 nM isoproterenol in intracellular solution.

### Cardiomyocyte Shortening, Ca^2+^ Transient (CaT), and Sarcoplasmic Reticulum (SR) Load

Cell shortening, CaT, and SR load were detected using IonOptix system [18] from freshly isolated cardiomyocytes from control and DIC-treated mice. Cell contraction was measured using a high-speed video camera to record sarcomere movement. The sarcomere was used to calculate the sarcomere length using the FFT algorithm. For CaT and SR load experiments, cells were loaded with Fluo-4 and paced at 10 V at a frequency of 0.5 Hz. To induce maximum sarcoplasmic reticulum, 20 mM caffeine was applied.

### Flow Cytometry Analyses of Cardiac Fibroblasts and Cardiomyocytes

Isolated cardiac cells from control and DIC-treated mice were filtered through 200-µm cell strainer, re-suspended in Ca^2+^ and Mg^2+^ free phosphate-buffered saline (PBS), fixed with 0.4% paraformaldehyde, before treating with antibodies.

### Plasma Cytokine Levels

Plasma cytokine levels from samples collected 4 weeks after DIC treatment were analyzed using a Cytometric Bead Array kit and FCAP Array software following the manufacturer's protocol [19].

### Metabolomic Profiling of Oxylipins

Oxylipin profiling was performed using a modification of a previously published method [19, 20].

### Statistical Analysis

All data are reported as mean ± standard error, unless otherwise stated. Statistical comparisons were analyzed by Student’s *t*-tests or one-way ANOVA followed by Bonferroni tests for post hoc comparison using GraphPad Prism version 9 (San Diego, CA). Multivariate statistical analysis was performed using MetaboAnalyst version 5.0 to analyze the distinct clustering among groups. Oxylipins were represented on a heat map using Euclidean distance and Ward’s clustering. Unsupervised principal component analysis (PCA) was performed to identify possible trends. Partial least-squares discriminant analysis (PLS-DA) was applied to test the differences between control and DIC-treated groups. Validation of the model was performed using *R*^2^ and *Q*^2^ values derived from cross-validation algorithm. Statistical significance was considered to be achieved when *P* < 0.05.

## Results

### Chronic DIC-Treated Mice Exhibited Impaired Cardiac Function

**Fig. 1:**
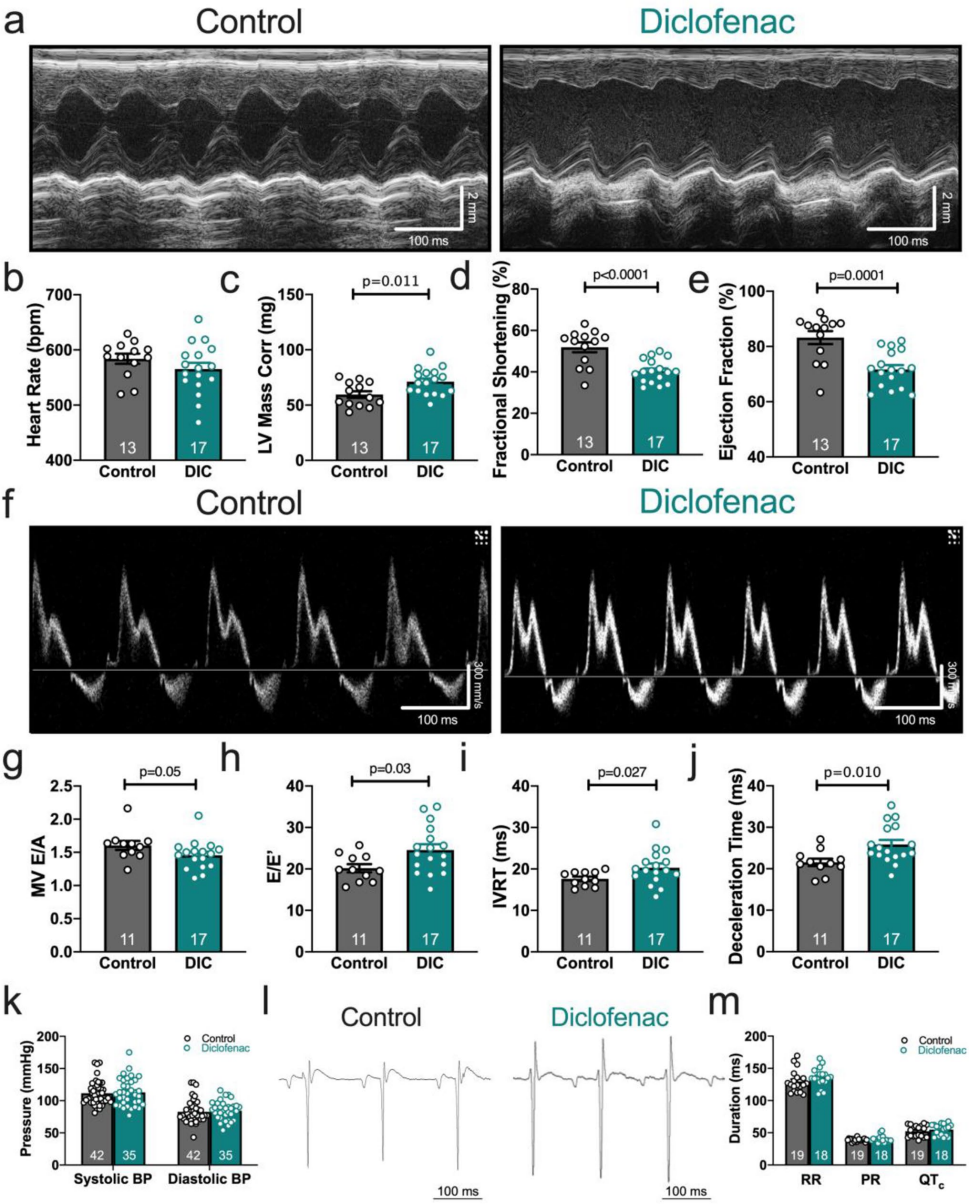
DIC-treated mice exhibited impaired cardiac function. After 4 weeks, mice were subjected to cardiovascular analyses. **a** Representative M-mode images at the parasternal short axis are shown. **b** Heart rate did not vary between the two groups in conscious echocardiography recordings. **c** Structurally, left ventricular mass (corrected) was significantly higher in DIC-treated mice. **d** Additionally, DIC-treated mice exhibited depressed systolic function, as evident by the reduced fractional shortening and **e** ejection fraction. **f** Blood flow velocity through the mitral valve was utilized to assess diastolic function as shown in the representative images. **g** With DIC treatment, the mitral valve E/A ratio was significantly lower. **h** The E/E′ ratio, **i** isovolumentric relaxation time (IVRT), and **j** deceleration time were significantly prolonged. **k** Blood pressure, as measured by tail cuff pressure, was not altered with DIC treatment. **l** Electrocardiography recordings, as seen in the representative images, and **m** quantitatively with analyses of RR, PR, and QT_c_, did not show any difference between DIC-treated mice and control mice. Data represented as mean ± SEM. Statistical significance was considered to be achieved when *P* < 0.05 by *t*-test

Recent evidence has suggested that inhibition of COX induces oxidative stress [9]. Since the heart relies on a dense network of mitochondria to supply energy, elevated oxidative stress is especially detrimental to the constantly beating heart. To investigate whether chronic DIC treatment affects cardiovascular function, mice were treated with (15 mg/kg/day) DIC for 4 weeks and subsequently monitored. The dosage was selected based on prior studies [21, 22]. Whole heart images showed no evidence of cardiac hypertrophy or chamber dilatation with no significant differences in the heart weight/body weight ratios between chronic exposure group and control (Figures S1A-D). However, there was a significant increase in mortality in the DIC-treated group (Figure S1E). Of those mice that survived, echocardiographic measurements revealed severely depressed cardiac function. Representative M-mode tracing at the parasternal short axis is shown for the two groups (Fig. 1a). Recordings were performed in conscious mice to eliminate the effects of volatile anesthetic [23]. There were no significant changes in heart rate in the chronic exposure group (Fig. 1b); however, left ventricular mass was significantly higher in DIC-treated mice, with a concomitant decrease in fractional shortening and ejection fraction (Fig. 1c–e). Diastolic function was also assessed by monitoring blood flow velocity through the mitral valve (MV) during early (E wave) and late diastole (A wave) as shown in the representative images in Fig. 1f. With treatment, there was a decline in the MV E/A ratio (Fig. 1g) with a prolongation of the E-wave deceleration time (Fig. 1h) and isovolumetric relaxation time (Fig. 1i). Together, these data suggest that chronic DIC treatment impaired both systolic and diastolic function.

Additionally, NSAIDs have been shown to elevate blood pressure [24] and increase the risk of cardiac arrhythmias [3, 25]. We measured systolic and diastolic blood pressure using tail cuffs. Mice were acclimated with the procedure 5 days prior to blood pressure recordings. Training was done using positive reinforcement. Under our treatment regimen, chronic treatment did not affect systolic and diastolic blood pressure (Fig. 1j). In addition, we recorded ECGs in anesthetized mice after 4 weeks of treatment. Representative ECG traces are shown in Fig. 1k. There were no significant differences in RR, PR, and QT_c_ intervals (Fig. 1l). Taken together, our data suggest that chronic exposure to DIC depressed cardiac contractile function, but did not affect systemic blood pressure or heart rate or rhythm.

### Mechanistic Underpinnings of the Observed Systolic and Diastolic Dysfunction in Chronic DIC Exposure Mice, Revealed Using Single-Cell Shortening and Ca^2+^ Transients Analyses

**Fig. 2:**
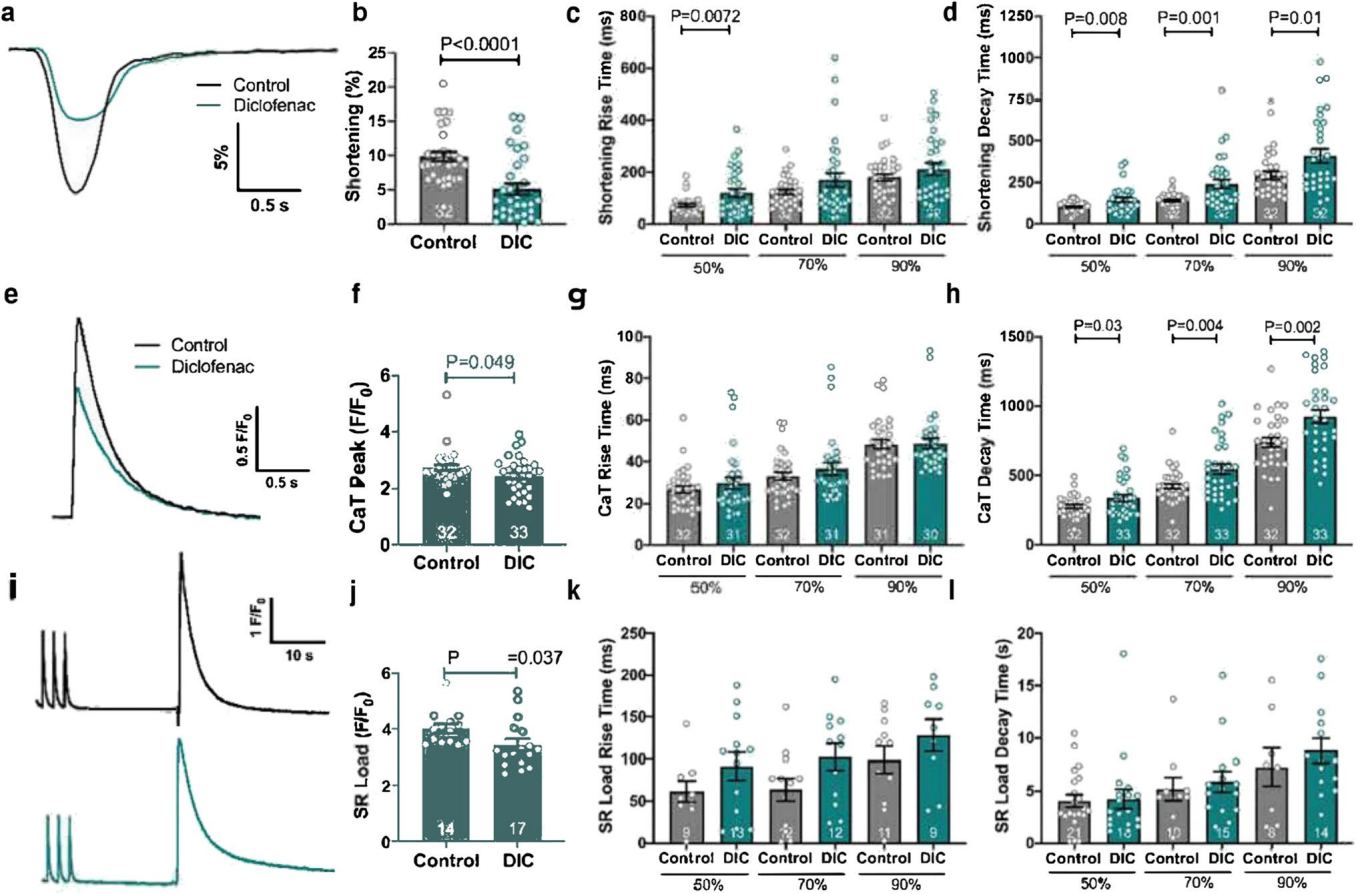
DIC treatment reduced sarcomere shortening, Ca^2+^ transient, and SR load in isolated cardiomyocytes. Cardiomyocytes were isolated from both groups and loaded with Fluo-4 to monitor Ca^2+^ dynamics. **a** Cardiomyocyte shortening was significantly impaired with DIC treatment, as manifested in the representative traces. **b** The percentage of shortening was reduced, **c** while the rise time at 50% **d** and shortening decay time at 50%, 70%, and 90% were prolonged. **e** Representative traces of Ca^2+^ transients are shown for both groups. **f** With treatment, there was a decrease in peak Ca^2+^ transient amplitude. **g** Although the time it took to reach the peak was not different, **h** there was a significant prolongation of the Ca^2+^ transient decay time at 50%, 70%, and 90%. **i** Additionally, the sarcoplasmic reticulum load was decreased, as shown in the representative images and **j** a significant decrease in SR Ca^2+^ load in the chronic exposure group. **k** and **l** There was no significant differences in SR Ca^2+^ load rise time and the decay time between the two groups. Data expressed as mean ± SEM. *n* = 5–6 mice for each group. Statistical significance was considered to be achieved when *P* < 0.05 by *t*-test

To determine the mechanisms underlying the observed systolic and diastolic dysfunction in the chronic DIC-treated group, we took advantage of single-cell analyses to quantify sarcomere shortening and Ca^2+^ dynamics using IonOptix system. In corroboration with our echocardiography data, cardiomyocytes isolated from DIC-treated mice exhibited reduced cell shortening as shown in Fig. 2a. Quantitatively, percentages of cell shortening were significantly decreased with treatment (Fig. 2b, *p* < 0.0001). Moreover, the time to reach 50% of the peak amplitude (Fig. 2c) and the decay time to 50%, 70%, and 90% from peak amplitude were significantly prolonged in treated cardiomyocytes (Fig. 2d).

Since Ca^2+^ plays a pivotal role in mediating cellular contractility, we examined Ca^2+^ transients and the sarcoplasmic reticulum (SR) Ca^2+^ load. Representative traces of Ca^2+^ transient recordings are shown in Fig. 2e. There was a significant decrease in the peak amplitude of the Ca^2+^ transients in DIC-treated cardiomyocytes (Fig. 2f). Although there were no changes in the time-to-peak amplitude in treated cardiomyocytes (Fig. 2g), the decay time was significantly prolonged in the treated cardiomyocytes (Fig. 2h). To acquire the SR Ca^2+^ load, we applied caffeine to induce maximal release of Ca^2+^ from the SR, as shown by the representative traces in Fig. 2i–j. Chronic DIC treatment significantly decreased the SR Ca^2+^ load (Fig. 2j). However, there were no changes in the kinetics in the presence of caffeine (Fig. 2k-l), suggesting no significant changes in the Na^+^-Ca^2+^ exchange activities. Therefore, the significant decrease in SR Ca^2+^ load is likely a result of a decrease in SR Ca^2+^-ATPase (SERCA) activities, a well-described remodeling of Ca^2+^ cycling proteins in cardiac hypertrophy and failure [26]. Together, our data demonstrate that chronic DIC treatment significantly reduced SR Ca^2+^ load, leading to a reduction in Ca^2+^ transients and single-cell shortening as well as prolongation of the decay kinetics of both sarcomere shortening and Ca^2+^ transients (Fig. 2c and h). The net effects are systolic and diastolic dysfunction as we have demonstrated at the in vivo level.

### Chronic DIC Treatment Increased Cardiac Fibroblast Proliferation

**Fig. 3:**
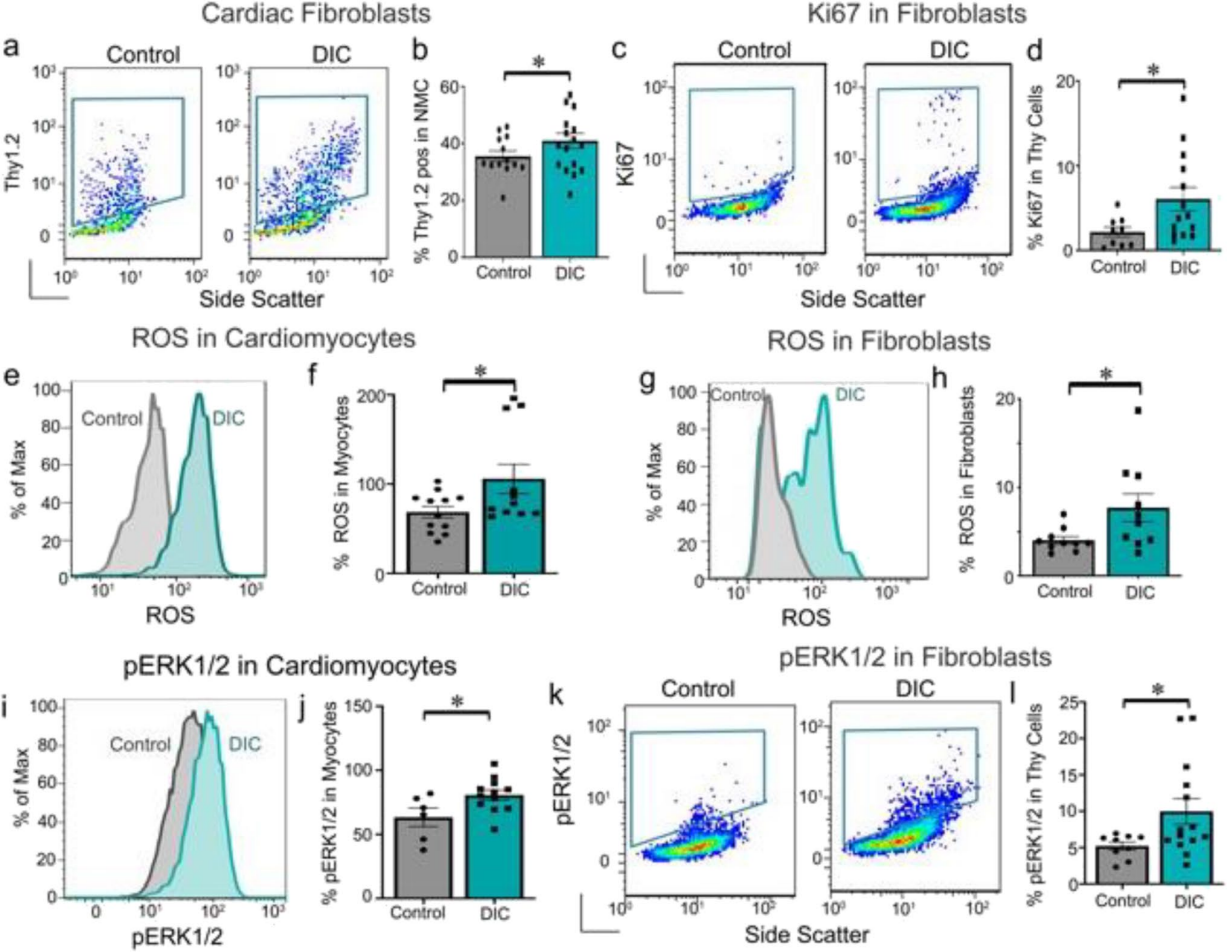
DIC treatment increases cardiac fibroblast proliferation and oxidative stress. **a** Flow cytometric analysis of the increase percentage of cardiac fibroblasts in the DIC-treated mice compared to control mice. **b** Summary data from **a**. *n* = 13–18 mice per group. **c** Flow cytometric analysis showing the increase proliferation of cardiac fibroblasts with DIC treatment. **d** Summary data from **c**. *n* = 9–14 mice per group. **e** and **f** Increased oxidative stress in cardiomyocytes **g** and **h** and fibroblasts as assessed by ROS from mice treated with DIC compared to control mice. *n* = 9–13 mice per group. **i** and **j** Increase in pERK1/2 in cardiomyocytes and **k** and **l** fibroblasts from DIC-treated and control groups. Statistical significance was considered to be achieved when *P* < 0.05 by *t*-test. Representative results are shown. *X* and *Y* axes represent arbitrary units. Data expressed as mean ± SEM.

Since DIC has been associated with increase cardiovascular risk [3, 27], and we found that DIC treatment resulted in impaired systolic and diastolic cardiac function, we investigated structural changes in the heart. Cardiac fibroblasts are the most important contributors of the collagen matrix deposition. To evaluate structural changes, cardiac sections from control and DIC-treated mice were stained with Masson’s Trichrome (Figure S1F). Since collagen staining was not evident in the cardiac sections, increase in cardiac fibroblasts was quantified using flow cytometry from isolated cardiac cells. We have previously characterized cardiac fibroblasts as cells expressing Thy1.2^+^/Lin^−^/CD45^−^/CD31^−^ using flow cytometry [19]. When digested single cells from control and DIC-treated mice were analyzed by flow cytometry, we saw a significant increase in percentages of fibroblasts in the DIC-treated group compared to the control (Control 36 ± 1%, DIC 42 ± 1%, Fig. 3a–b). We further utilized Ki67 to analyze the proliferative capacity among cardiac fibroblasts with DIC treatment. There was a significant increase in the percentage of Ki67 in the DIC-treated fibroblasts compared to control (Control 2 ± 0.5%, DIC 6 ± 1%, Fig. 3c–d).

### Chronic DIC Treatment Increased Oxidative Stress in Fibroblasts and Cardiomyocytes

Exposure to DIC has been shown to increase oxidative stress. Increased levels of ROS lead to dysfunction of multiple biological signaling pathways[16, 21]. Therefore, we quantified the level of ROS in cardiac cells, specifically cardiomyocytes and fibroblasts. Analysis of cardiomyocytes from the DIC-treated and control groups revealed a significant increase in ROS in DIC-treated cardiomyocytes compared to control cardiomyocytes (Fig. 3e–f). We also demonstrate a significant increase in ROS in DIC-treated fibroblasts compared to control fibroblasts (Fig. 3g–h).

Increase in ROS has been shown to activate members of the mitogen-activated protein kinase (MAPK) signaling cascade [21]. Therefore, we investigated the activation of downstream members of the MAPK cascade, the extracellular signal-regulated kinase 1 and 2 (ERK1/2) in fibroblasts and cardiomyocytes. Here, there was a significant increase in pERK1/2 in DIC-treated cardiomyocytes (Fig. 3i–j). Similarly, there was a significant elevation in the levels of phosphorylated ERK1/2 (pERK1/2) in fibroblasts (Control 5 ± 0.5% vs. DIC 10 ± 1%, Fig. 3k–l).

### Chronic DIC Treatment Impaired Mitochondrial Function

**Fig. 4:**
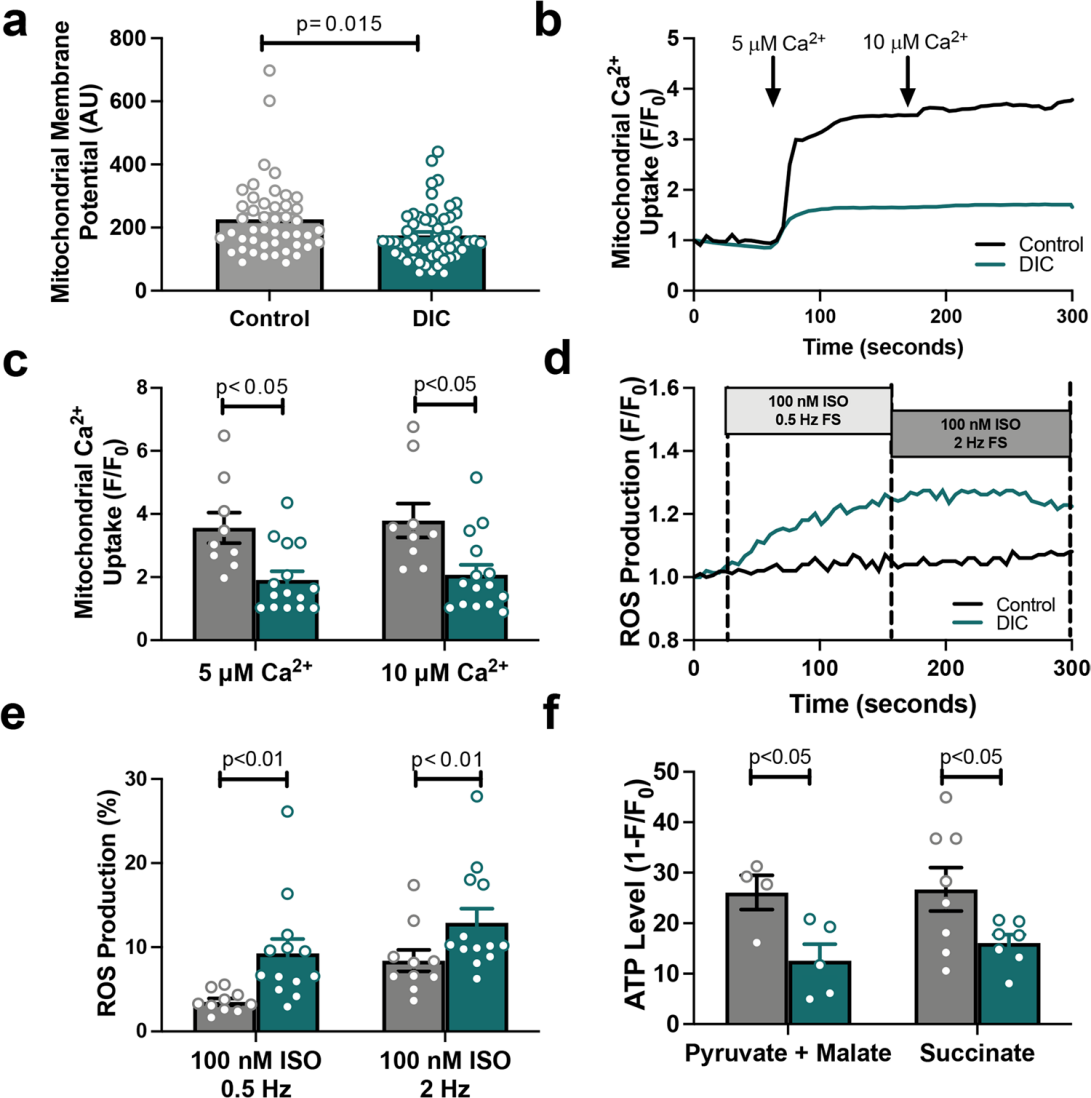
Chronic DIC treatment impaired mitochondrial function. Mitochondrial functional assessment was done in isolated cardiomyocytes. **a** Mitochondrial membrane potential was monitored using TMRM. With DIC treatment, mitochondrial membrane potential became more depolarized, relative to control. **b** Representative traces show that mitochondrial Ca^2+^ uptake was reduced in isolated cardiomyocytes from treated mice. **c** Quantitatively, mitochondrial Ca^2+^ was significantly lower at external [Ca^2+^] of 5 μM and 10 μM in treated cardiomyocytes. **d** To see the effects of ROS production from the mitochondria, cardiomyocytes were stimulated with both field stimulation and isoproterenol, as shown in the representative traces. **e** In both conditions, ROS production was greater in treated cardiomyocytes. **f** Moreover, ATP level was significantly reduced in treated cardiomyocytes after Complex I and Complex II mediated respiration. Data expressed as mean ± SEM. *n* = 3–6 mice for each group. Statistical significance was considered to be achieved when *P* < 0.05 by *t*-test and repeated measures *t*-test

As an energetically demanding organ, the heart is densely packed with mitochondria. Proper communication with these mitochondria is crucial for sufficient energy production on a beat-to-beat basis [28]. To determine the effects of DIC treatment on mitochondrial function, we examined mitochondrial membrane potential, Ca^2+^ uptake, ROS production, and ATP level. We demonstrate that with DIC treatment, mitochondrial membrane potential was significantly depolarized (Fig. 4a). Additionally, permeabilized DIC-treated cardiomoycytes exhibited reduced mitochondrial Ca^2+^ uptake when challenged with 5 and 10 μM of Ca^2+^, as shown in the representative traces and quantitatively (Fig. 4b–c). Since ROS is known to increase after β-adrenergic receptor stimulation and cellular stress, [29] we subjected cardiomyocytes to both isoproterenol (ISO) and field stimulation (FS). With 100 nM ISO and 0.5 Hz FS, we observed a significant increase in ROS in chronic DIC-treated cardiomyocytes relative to control, as depicted in Fig. 4d. Even with an increase in FS to 2 Hz, treated cardiomoycytes still exhibited higher ROS generation relative to control (Fig. 4e). Since reduced mitochondrial Ca^2+^ uptake and elevated ROS generation can impair energy production, [30] we directly monitored the ATP level after Complex I (5 mM pyruvate/5 mM malate) and Complex II (5 mM succinate) mediated respiration. With Complex I substrates, the ATP generation was significantly lower in the treated cardiomyocytes (Fig. 4f). This was also seen after using Complex II substrate. Together, these data suggest that mitochondrial function was significantly impaired, at least in part, due to a significant increase in ROS generation, that may further contribute to an increase in mitochondrial oxidative stress.

### Chronic DIC Treatment Alters the Oxylipin Profile and Increases Inflammatory Cytokines

**Fig. 5:**
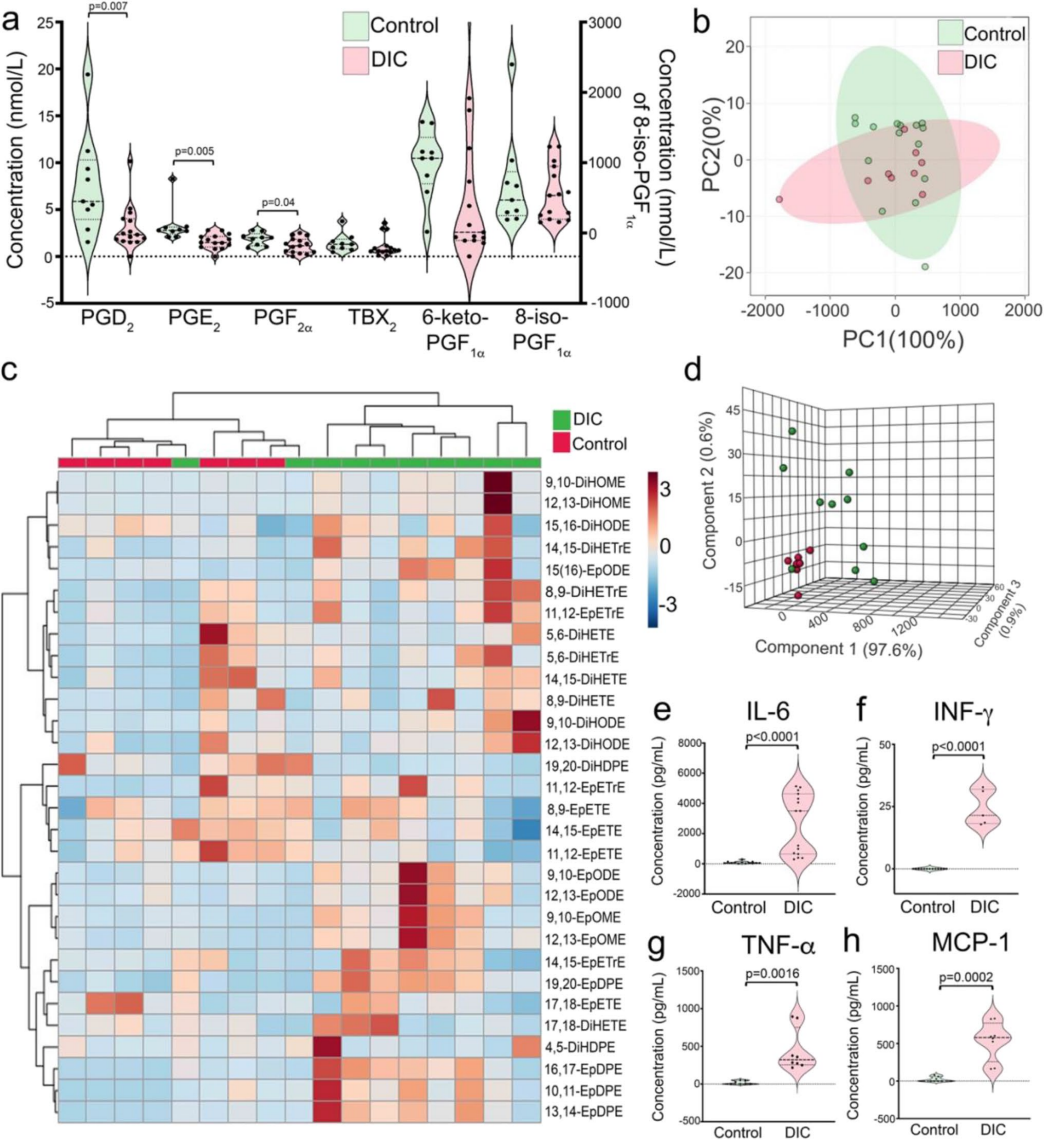
Chronic DIC treatment alters the oxylipins and increases inflammatory cytokines. **a** Plasma oxylipin concentration of prostanoids from DIC-treated and control groups. **b** Principal component analysis comparing prostaglandin concentrations between DIC-treated and control groups. **c** Heat map representing ARA, EPA, DHA, LA, and ALA metabolites post normality analysis. The color scale from − 3 to 3 represents the *z*-score. A positive *z*-score (increase in red color intensity) reflects increased metabolite concentration and a negative *z*-score (increase in blue color intensity) reflects decreased concentration. Red and green colored boxes on top of the heatmap represent the two groups (red = DIC treatment and green = control group) with their position showing how they cluster together, as determined by hierarchical clustering using Ward’s algorithm. **d** Partial least-squares discriminant analyses comparing metabolites from **c**. **e**–**h** Serum concentration of inflammatory cytokines and chemokine. Data expressed as mean ± SEM. *n* = 10–16 mice for each group. Statistical significance was considered to be achieved when *P* < 0.05 by *t*-test or one-way ANOVA with post hoc Bonferrori’s test

Polyunsaturated fatty acids including ARA, eicosapentaenoic acid (EPA), docosahexaenoic acid (DHA), linoleic acid (LA), and α-linolenic acid (ALA) can be metabolized via three pathways by the *COX*, *LOX*, and *CYP450* enzymes. Since these three enzymatic pathways share the same substrates, to determine the underlying mechanism of the cardiotoxicity with chronic exposure to DIC, we analyzed the oxylipin metabolites from ARA, EPA, DHA, LA, and ALA, many of which are targeted by DIC. Sixty oxylipins were analyzed by ultrahigh-pressure LC/MS/MS from chronic DIC-treated and control plasma samples [19]. DIC-treated mice showed a significant decrease in prostanoids (PGD_2_, PGE_2_, PGF_2α_, TXB2, 6-keto-PGF_1α_, and 8-iso-PGF_1α_), consistent with the COX enzymes targeting effects of DIC (Fig. 5a). Unsupervised PCA revealed a separation of samples based on DIC treatment and control groups (Fig. 5b).

Indeed, a hierarchical cluster analysis using Ward’s algorithm showed clustering of metabolites from the DIC-treated group, that was separated from clustering of metabolites from the control group (Fig. 5c, green-DIC and red-control). To further explore these differences, a PLS-DA was performed. This resulted in a four-component model with a goodness of prediction *Q*^2^ of 0.28 and a goodness of fit *R*^2^ of 0.71 (Fig. 5d).

Of interest in cardioprotection is the metabolism of ARA by *CYP450* enzymes to epoxyeicosatrienoic acidsepoxyeicosatrienoic acids (EETs), that are known to anti-inflammatory metabolites. However, EETs are further metabolized into dihydroxyeicosareinoic acid (DHETs) with diminished cardioprotective activities. Therefore, to understand the effect of DIC treatment on ARA and *CYP450* pathway, we analyzed the EETs/DHETs ratio. Our analysis demonstrated a significant decrease in the 14,15-EET/DHET ratio in the DIC-treated groups compared with control group (Figure S2A). Moreover, metabolites of the *LOX* pathways demonstrated a modest difference between the two groups (Figure S2B).

While DIC suppresses inflammation, chronic DIC treatment is associated with increased levels of inflammatory cytokines including TNF-α [22]. Our data demonstrate a significant increase in proinflammatory cytokine and chemokine levels including interleukin 6 (IL-6), interferon-γ (IFN-γ), TNF-α, and monocyte chemoattractant protein-1 (MCP-1) in the DIC-treated mice compared to controls (Fig. 5e–h).

## Discussion

The widespread availability, usage, and improper disposal of NSAIDs are not only detrimental to the environment [2], but also harmful to humans [24]. The cardiotoxic effects of NSAIDs vary among individual drug and also is dose-dependent. In the current study, we demonstrate that 4 weeks of chronic exposure to DIC, a commonly prescribed NSAID, and in terms of tonnage, one of world’s major medication, result in impaired cardiac systolic and diastolic function (Fig. 1), as well as increased cardiac fibroblast proliferation (Fig. 3). Although the risk of cardiovascular complications such as blood pressure elevation [24] and arrhythmias [3, 25] is higher in patients who take NSAIDs, we did not observe any differences in blood pressure or any arrhythmias in the mice that were treated relative to control mice. This is likely attributed to the regimen chosen. However, the 4-week treatment plan was chosen to investigate DIC-induced cardiotoxicity, since we observed an increase in mortality of mice with this schedule (Figure S1B). Further investigation demonstrated that chronic DIC treatment severely affected the digestive system, manifested by ischemic colitis in treated mice that did not survive the 4-week treatment regimen. The effect of NSAIDs on the gastrointestinal tract (GI) is due to the unique local environment, the pharmacokinetics of the drugs, and mitochondrial dysfunction. The effects in the GI tract may result in high mortality in mice treated with DIC and not cardiac dysfunction alone. The decreased levels of prostanoids, in particular PGE_2_ with COX inhibition, lead to removal of the protection in the GI, leading to gastric ulcers and erosion of gastric cells [31]. The accumulation of NSAIDs in the GI lining independently causes mitochondrial dysfunction, leading to GI damage [32].

### Diclofenac and Ca^2+^ Handling

Ca^2+^ handling is tightly regulated for normal cardiac function. Since Ca^2+^ is an important regulator that mediates an array of cardiac signaling pathways [33], alterations in Ca^2+^ can result in severe cardiac consequences [34]. Indeed, one primary role of Ca^2+^ in the heart is to couple energy production with energy demand [35], which involves proper Ca^2+^ communication between the SR and the mitochondria at microdomains [36]. It has been reported that DIC inhibits L-type Ca^2+^ channels in cardiomyocytes [37], which likely affects Ca^2+^-induced Ca^2+^ release from the SR. Indeed, our in vitro findings showed isolated cardiomyocytes from treated mice exhibited impaired Ca^2+^ transient peak amplitude and kinetics, as well as depressed sarcoplasmic reticulum (SR) load (Fig. 2).

It has been shown that Ca^2+^ entry into the mitochondria is crucial for activating key dehydrogenases, which subsequently increases production of reduced NADH and FADH_2_ to further fuel oxidative phosphorylation [38]. Along with the reduced Ca^2+^ transient peak amplitude, we found depressed mitochondrial Ca^2+^ uptake when challenged with increasing concentrations of external Ca^2+^, which suggests impaired communication with the SR and mitochondria with chronic DIC treatment (Fig. 4). Additionally, our data demonstrate that chronic DIC treatment reduced ATP levels after Complex I and Complex II mediated respiration in isolated cardiomyocytes. Although a reduction in Ca^2+^ transient, SR load, and reduced ability of mitochondria to uptake Ca^2+^ contribute to lower energy production, ROS production can also impair mitochondrial function.

### Diclofenac and Oxidative Stress

DIC has been shown to increase the production of ROS in several cell types including mouse neonatal cardiomyocytes [21]. Members of the MAPK signaling pathway induce cardiac fibrosis by activating the proliferation and collagen production of cardiac fibroblasts [39]. Specifically, an increase of ERK1/2 activity activated by increased ROS enhances cardiac fibroblast proliferation and thereby cardiac fibrosis and dysfunction [19, 20]. In response to increased ROS, fibroblasts can produce proinflammatory chemokines and chemokines, leading to exacerbation of cardiac dysfunction. Our study shows a significant increase in ROS and a concomitant increase of activated ERK1/2 in cardiomyocytes and fibroblasts from mice treated with DIC (Fig. 3). The elevated ROS levels and the activation on ERK1/2 in our study suggest that increase in cardiac fibroblast proliferation was partly due to the activation of the MAPK signaling cascade initiated by ROS.

Furthermore, we found that mitochondrial membrane potential (ΔΨm) from DIC-treated mice was severely more depolarized than control mitochondria (Fig. 4), which suggests impaired mitochondrial function. A previous study has found that elevated levels in PGE_2_ can dissipate membrane potential, [40] which may partly explain our depolarized mitochondrial membrane potential. Similarly, a recent study by Barndolini et al., evaluating the effects of ketoprofen and DIC in immortalized human cardiomyocytes, demonstrates a decrease in mitochondrial potential. One possible underlying mechanism may be due to the opening of the mitochondrial permeability transition pores promoted by the Ca^2+^ accumulation in mitochondria. [16]

Indeed, mitochondria from DIC-treated mice were more susceptible to ROS generation under basal conditions and after β-AR stimulation and field stimulation. Mitochondrial permeability transition pores are stimulated by mitochondrial Ca^2+^ accumulation to facilitate the free passage of low molecular weight compounds between the inner mitochondrial matrix and the cytosol [16]. The underlying mechanism of DIC-dependent depolarization may in part be due to inefficient opening of the mitochondrial permeability transition pores, which is exacerbated by impaired mitochondrial Ca^2+^ uptake shown in our study.

It has been reported that DIC toxicity was evident in the ultrastructure of livers from chicks fed a DIC diet [41]. In addition to multiple structural alterations in the organelles, the authors noted the prevalence of diminished mitochondrial membrane integrity. These structural alterations due to DIC toxicity can lead to impaired mitochondrial function, as supported in our study. Furthermore, another study found that glutathione (GSH), a necessary component in the cellular antioxidant machinery, is able to mitigate some of DIC-induced inhibitory effects of ATP production in rat liver mitochondria [42]. In alignment with our study, we found that in addition to decreased ATP levels with DIC treatment, there was also a significant increase in ROS generation. Our data therefore strongly suggest that mitochondrial function was overall impaired due to chronic DIC treatment, in part, by an increase in oxidative stress.

### Important Insights into the Mechanisms Underlying Mitochondrial Dysfunction from Chronic DIC Exposure Using Metabolomic Analyses

Polyunsaturated fatty acids including ARA, EPA, DHA, LA, and ALA can be metabolized via three broad pathways governed by *COX*, *LOX*, and *CYP450* enzymes to yield prostaglandins, leukotrienes, EETs, and hydroxyeicosatetraenoic acid (HETE) [43]. The imbalance arising due to *COX* inhibition between prostaglandins (anti-thrombogenic) and thromboxanes (thrombogenic) is one of the main causes of thrombotic events, which increases the cardiovascular risk with *COX* inhibition [44]. However, DIC not only inhibits the *COX-1* and *COX-2* enzymes, but also affects other pathways including *LOX* and *CYP450* leading to a decrease in the cardioprotective anti-inflammatory metabolites [44]. We have extensively shown that EETs, the *CYP450* metabolites, are anti-inflammatory and cardioprotective in preventing ventricular hypertrophy, electrical remodeling, and cardiac fibrosis and reducing both atrial and ventricular arrhythmia inducibility in cardiac hypertrophy and MI models [19, 20, 45]. Our results in this study demonstrate a decrease in EETs isomers with DIC treatment (Figure S3A) suggesting a reduction in cardioprotection with DIC treatment.

HETEs (12-HETE and 15-HETE), which are metabolized through the *LOX* pathway, cause mitochondrial dysfunction by decreasing mitochondrial respiration and transmembrane potential [46]. An increase in HETEs (8-, 9-, 12- and 15-HETEs, Figure S3B) and a more depolarized mitochondrial potential with DIC treatment in our study suggest that DIC increases mitochondrial ROS through modifications in oxylipin levels. It has been shown that mitochondrial ROS not only activates PGE synthase, which provides substrate for *COX-1* and *COX-2*, but also increases *COX-2* gene expression [47, 48]. Thus, paradoxically, DIC induces the production of *COX* in multiple ways; the same enzyme that DIC is used to inhibit.

### Mitochondrial Oxidative Stress, Inflammatory Mediators, and Cardiac Fibrosis

Mitochondrial oxidative stress and inflammation have both been implicated in the development of cardiac dysfunction leading to chronic heart failure[49]. The mitochondrial electron transport chain is a predominant source of intracellular ROS. Mitochondrial ROS activate the release of molecules known as danger-associated molecular patterns (DAMPs), that directly trigger the production of proinflammatory cytokines through the activation of inflammasome, leading to cardiac dysfunction[50]. The critical roles of mitochondrial ROS in the induction of cardiac fibrosis and hypertrophy leading to heart failure have been demonstrated [12]. In our study, we demonstrate that chronic exposure of DIC increases mitochondrial ROS production and cardiac fibroblast proliferation and activation. Future studies are needed to determine a direct causal effect of increased mitochondrial ROS production on cardiac fibrosis, leading to cardiac dysfunction by chronic DIC exposure.

## Conclusions

We demonstrate that chronic treatment with DIC results in impaired cardiac function. In corroboration with this finding, our in vitro data in isolated cardiomyocytes show reduced cell shortening and impaired Ca^2+^ dynamics at the cellular level. With DIC treatment, mitochondrial membrane potential becomes depolarized and ATP production is diminished. Moreover, cardiomyocytes exhibit increased ROS and mitochondria from DIC-treated mice show increased susceptibility to ROS generation after application of a β-AR agonist along with field stimulation. Taken together, our data suggest that chronic DIC treatment results in elevated oxidative stress and altered oxylipin profiles towards inflammatory features, which contributed to mitochondrial dysfunction and ultimately impaired cardiac function.

## Supplementary Information

Below is the link to the electronic supplementary material.

Supplementary file1 (DOCX 7072 KB)

## Data Availability

All data generated or analyzed during this study are included in this published article and its supplementary information files.

## Code Availability

Commercially available GraphPad Prism version 9 (San Diego, CA) and online MetaboAnalyst version 5.0 were used.
