# Supplementary material for: Chronic Diclofenac Exposure Increases Mitochondrial Oxidative Stress, Inflammatory Mediators, and Cardiac Dysfunction
Source: Cardiovasc Drugs Ther. 2021 Sep 9;37(1):25–37. doi: 10.1007/s10557-021-07253-4 (PMC8904649; doi:10.1007/s10557-021-07253-4)
Supplement: Supplementary file 1 — Supplementary file1 (DOCX 7072 KB) [file 10557_2021_7253_MOESM1_ESM.docx]

**Supplemental Information for:**

**Chronic Diclofenac Exposure Increases Mitochondrial Oxidative Stress, Inflammatory Mediators, and Cardiac Dysfunction**

Phung N. Thai^1^, Lu Ren^1^, Wilson Xu^1^, James Overton^1^, Valeriy Timofeyev^1^, Carol E. Nader^1^, Michael Haddad^1^, Jun Yang^2^, Aldrin Gomes^3^, Bruce D. Hammock^2^, Nipavan Chiamvimonvat^1,4,5^, Padmini Sirish^1,5^

^1^Department of Internal Medicine, Division of Cardiovascular Medicine, University of California, Davis

^2^Department of Entomology, University of California, Davis

^3^Department of Physiology and Membrane Biology

^4^Department of Pharmacology, University of California, Davis

^5^Department of Veterans Affairs, Northern California Health Care System, Mather, CA

Corresponding Authors:

Padmini Sirish, Ph.D. and Nipavan Chiamvimonvat, M.D.

Department of Internal Medicine, Division of Cardiovascular Medicine

University of California, Davis

451 Health Science Drive, GBSF 6315

Davis, CA 95616

Department of Veterans Affairs, Northern California Health Care System

10535 Hospital Way Mather, CA 95655

Email: [psirish@ucdavis.edu](mailto:psirish@ucdavis.edu) and [nchiamvimonvat@ucdavis.edu](mailto:nchiamvimonvat@ucdavis.edu)

**The PDF includes:**

1. Supplementary text for SI Materials and Methods
2. Figs. S1 and S2
3. References for SI reference citations

**DATA SUPPLEMENT**

**Supplemental Methods and Materials:**

**SI Materials and Methods**

The investigation conforms to the Guide for the Care and Use of Laboratory Animals published by the US National Institutes of Health (NIH Publication No. 85-23, revised 1996) and was approved by the University of California, Davis Institutional Animal Care and Use Committee.

***Echocardiography:***

8- to 12- week old male and female C57BL/6 mice were treated with DIC (15 mg/kg/day) or vehicle alone in drinking water for four weeks and subsequently monitored in accordance with approved protocols of the IACUC Committee at the University of California, Davis. Echocardiography was performed using the Visualsonics Vevo 2100 ultrasound system (Fujifilm VisualSonics Inc., Toronto, Canada) with the MS 550D probe. Cardiac function was assessed after 4 weeks of either saline or DIC treatment. Systolic function (B-mode videos and M-mode images) was measured in conscious mice and diastolic function (pulse-wave Doppler) was measured in the presence of 0.5%-1% isoflurane. Analysis was performed by investigators blinded to the treatment groups.

***Blood Pressure Recordings:***

Mice were acclimated to the non-invasive, tail vein blood pressure recording system (Kent Scientific Corporation, Torrington, CT) for five days before actual measurements were taken. Positive reinforcement was given to reduce sympathetic drive and ensure reproducibility. Measurements were taken in accordance with manufacturer’s recommendations. A total of 15 blood pressure recordings were captured for each mouse, and the last 7 blood pressure recordings were used for analysis. Analysis was performed in a blind fashion.

***Mitochondrial Membrane Potential:***

Mitochondrial membrane potential was assessed in freshly isolated control and DIC-treated cardiomyocytes using tetramethylrodamine ester (TMRM, ThermoFisher Scientific; λ_ex_ = 514 nm, λ_em_ = 590 nm) [1]. Cardiomyocytes were incubated with 5 nM TMRM for 30 minutes at 37°C. All recording solutions contained 5 nM TMRM. Analysis was performed in a blinded fashion using ImageJ FIJI Software

***Mitochondrial Ca^2+^ Uptake:***

Mitochondrial Ca^2+^ Uptake was monitored in freshly isolated control and DIC-treated cardiomyocytes using X-Rhod-1 AM (Thermo Fisher Scientific, Waltham, MA) [1]. Cells were loaded with X-Rhod-1 AM for 40 minutes at 37°C. Cells were initially perfused with 50 µg/ml of Saponin in intracellular solution containing (in mM): 135 KCl, 0 NaCl, 20 HEPES, 5 pyruvate, 2 glutamate, 2 malate, 0.5 KH_2_PO_4_, 0.5 MgCl_2_, 15 2,3-butanedione monoxime, 5 EGTA, and 1.86 CaCl_2_ to yield a free [Ca^2+^]_i_ of 100 nM with pH 7.2. The solution was then switched to intracellular solution without saponin for baseline recording. Mitochondrial Ca^2+^ uptake was monitored by the change in fluorescence intensity, normalized to the baseline fluorescence intensity, after the addition of 5 μM Ca^2+^ and 10 μM Ca^2+^.

***Mitochondrial ATP Generation:***

ATP changes were monitored in freshly isolated control and DIC-treated cardiomyocytes using mag-fluo-4 AM (Thermo Fisher Scientific) [1]. Cardiomyocytes were loaded with 10 μM mag-fluo-4 AM (λ_ex_ = 488 nm, λ_em_ = 565-605 nm) for 30 minutes at 37°C. 5 mM pyruvate and 5 mM malate were using to stimulate Complex-I mediated respiration. 5 mM succinate was used to stimulate Complex-II mediated respiration.

***Cardiomyocyte Shortening, Ca^2+^ Transient (CaT), and Sarcoplasmic Reticulum (SR) Load:***

We used the IonOptix system (IonOptix LLC, Westwood, MA) to detect cell shortening, Ca^2+^ transient (CaT), and SR load [2] from freshly isolated cardiomyocytes from control and DIC-treated mice. Cell contraction was measured using a high-speed video camera (MyoCam-S) to record sarcomere movement. The sarcomere was used to calculate the sarcomere length using the FFT algorithm.

For CaT and SR load experiments, cells were loaded with 10 μM Fluo-4 for 30 minutes at room temperature (RT). The recording solution contained (in mM): 145 NaCl, 4 KCl, 1 CaCl_2_, 0.33 NaH_2_PO_4_, 1 MgCl_2_, 10 Glucose, 10 HEPES, at a pH = 7.4. Cardiomyocytes were paced at 10 V at a frequency of 0.5 Hz. To induce maximum sarcoplasmic reticulum, 20 mM caffeine was applied.

***Measurement of Plasma Cytokine Levels:***

Plasma samples were collected 4 weeks after DIC-treatment and stored at -70°C until assayed. Plasma cytokine levels were analyzed using a Cytometric Bead Array kit (CBA mouse inflammation kit, BD Biosciences). Briefly, thawed plasma samples were mixed for 2 hours at room temperature with florescence-labeled capture beads with the PE detection reagents to measure the concentrations of Interleukin-6 (IL-6), Interleukine-1β (IL-1β), Interleukin-10 (IL-10), Monocyte Chemoattractant Protein-1 (MCP-1), Tumor Necrosis Factor-α (TNF-α) and Interleukin-12p70 ( IL-12 p70). Samples were then washed with washing buffer and analyzed on a FACScan flow cytometer (BD Immunocytometry Systems). Data were analyzed using FCAP Array software V3.0 (BD Bioscience).

***Cardiomyocyte Isolation:***

Isolation procedure was performed according to the approved UC Davis Animal Care and Use protocol. Briefly, mice were injected with 0.1 ml heparin (1,000 units/ml) 10 minutes prior to heart excision. Mice were anesthetized with ketamine at 80 mg/kg and xylazine at 5 mg/kg intraperitoneally once before the cell isolation. Hearts were removed and placed in Tyrode’s solution (mmol l^−1^: NaCl 140, KCl 5.4, MgCl_2_ 1.2, *N*-2-hydroxyethylpiperazine-*N*′-2-ethanesulphonic acid (HEPES) 5 and glucose 5, pH 7.4). All chemicals were obtained from Sigma-Aldrich Chemicals (St. Louis, MO) unless stated otherwise. The aorta was cannulated under a dissecting microscope and mounted on a Langendorff apparatus. The coronary arteries were perfused in a retrograde fashion with Tyrode’s solution gassed with O_2_ at 37 °C for 3 minutes at a flow rate of about 3 ml/min. The solution was switched to Tyrode’s solution containing collagenase type 2 (1 mg/ml, 330 units/mg, Worthington Biochemical Corporation, Lakewood, NJ). After approximately 12 minutes of enzyme perfusion, hearts were removed from the perfusion apparatus and gently teased in high-K^+^ solution (mmol/L: potassium glutamate 120, KCl 20, MgCl_2_ 1, EGTA 0.3, glucose 10 and HEPES 10, pH 7.4 with KOH). Cells were then introduced to a Ca^2+^ ladder to ensure robustness of cells during functional recordings.

***Flow cytometric analysis of cardiac cells:***

Single cell suspensions were obtained from six groups of NSG mice as previously described.[3] The procedure was performed according to the approved UC Davis Animal Care and Use protocol. Briefly, mice were injected with 0.1 ml heparin (1,000 units ml^−1^) 10 min prior to heart excision, then anesthetized with pentobarbital intraperitoneally (80 mg kg^−1^). Hearts were removed and placed in Tyrode’s solution (mmol l^−1^: NaCl 140, KCl 5.4, MgCl_2_ 1.2, *N*-2-hydroxyethylpiperazine-*N*′-2-ethanesulphonic acid (HEPES) 5 and glucose 5, pH 7.4). All chemicals were obtained from Sigma-Aldrich Chemicals unless stated otherwise. The aorta was cannulated under a dissecting microscope and mounted on the Langendorff apparatus. The coronary arteries were perfused in a retrograde fashion with Tyrode’s solution gassed with O_2_ at 37 °C for 3 min at a flow rate of ~3 ml min^−1^. The solution was switched to Tyrode’s solution containing collagenase type 2 (1 mg ml^−1^, 330 units mg^−1^, Worthington Biochemical Corporation, Lakewood NJ). After ~12 min of enzyme perfusion, hearts were removed from the perfusion apparatus and gently teased in high-K^+^ solution (mmol l^−1^: potassium glutamate 120, KCl 20, MgCl_2_ 1, EGTA 0.3, glucose 10 and HEPES 10, pH 7.4 with KOH). Cells were filtered through 200 µm cell strainer, re-suspended in Ca^2+^ and Mg^2+^ free phosphate buffered saline (PBS), fixed with 0.4% PFA, before treating with phytoerythrin-conjugated anti-Thy1.2 (BD Bioscience, San Diego, CA), lineage antibody cocktail (CD3, CD14, CD19, CD20 and CD56, BD Bioscience), anti-CD45 (BD Bioscience), anti-CD31 (BD Bioscience, San Diego, CA), anti-troponin T antibody (ThermoFisher Scientific), anti-myosin heavy chain (Developmental Studies Hybridoma Bank, IA), Alexa fluor 488 anti-pERK1/2 (Cell Signaling), and proliferation-specific Ki67 antibody (15 μg ml^−1^, BD Bioscience) in PBS with donkey serum and 20 µg ml^-1^ DNAse-free RNAse (Sigma-Aldrich) overnight at 4°C. Cells were also stained with 40 µg ml^-1^ 7‐amino‐actinomycin D (7AAD, BD Bioscience, San Jose, CA) to measure the DNA content. Equal number of myocytes were isolated from control and DIC-treated hearts and 10,000 to 20,000 cells were analyzed in each run. Data were collected using a standard FACScan cytometer (BD Biosciences) upgraded to a dual laser system with the addition of a blue laser (15 mW at 488 nm) and a red laser (25 mW at 637 nm Cytek Development, Inc, Fremont, CA). Data were acquired using CellQuest software (BD Bioscience) and analyzed using FlowJo software (ver9.4 Treestar Inc., San Carlos, CA). Cells stained with isotype-matched IgG antibodies were used as controls to determine the positive cell population.

To measure oxidative stress, CellRox (Life Technologies, Carlsbad, CA) reagent was utilized according to the manufacturers protocol. The CellRox cell-permeable reagents are non-fluorescent while in a reduced state and upon oxidation exhibit strong fluorogenic signal. Apoptosis was measured using Annexin V (Invitrogen, Carlsbad, CA) conjugated to Alexa Fluor 647 according to the manufacturers protocol.

***Electrocardiography (ECG):***

ECG recordings were performed as previously described [4]. Briefly, mice were placed on a warming pad set to 37°C. Two ECG needles were placed subcutaneously adjacent to the mouse’s paws. Recordings were acquired using Bioamplifier (BMA 831, CWE, Inc., Ardmore, PA, USA). Signals were low-pass filtered at 0.2 kHz. Analysis for each animal was performed in a blinded fashion. The rate-corrected QT interval (QT_c_) was calculated using Bazett’s formula as reported by Mitchell et al. [5]

***Histological Analyses:***

Excised hearts were retrogradely perfused with PBS to wash out blood. Fixed hearts were embedded in paraffin, and serial sections of 5 μm in thickness were taken along the longitudinal axis and stained with Masson’s Trichrome to assess for collagen content.

***Metabolomic Profiling of Oxylipins:***

Plasma samples stored at -80 ºC were thawed at room temperature. Aliquots of plasma (200 µL) were spiked with a set of odd chain length analogs and deuterated isomers of several target analytes including hydroxyeicosatetraenoic acids (HETEs), prostaglandins, thromboxanes, epoxides (EpOMEs and EETs), and diols (DHOMEs and DHETs) contained in 10 µL of methanol, and then were extracted by solid phase extraction using Oasis HLB cartridges (Waters, Milford, MA). The HLB columns (1 cc, 60mg) were washed with 2 mL methanol and pre-conditioned with 2 mL water/methanol/acetic acid (95/5/0.1, v/v). Samples were then mixed with 200 µL of the pre-conditioning solution and loaded onto the column. The loaded column was then washed with 2 mL of the pre-conditioning solution and then dried for 5 min *in* *vacuo*. Target analytes were then eluted with 2 mL of ethyl acetate. The collected eluents were evaporated to dryness using a centrifugal vacuum concentrator and re-dissolved in 40 µL of methanol. The spiked samples were vortexed for 1 min, centrifuged at 14,000 r.p.m. for 5 min, and then transferred to analytical vials containing 150 µL inserts for analysis.

The oxylipin profiling was performed using a modification of a previously published method [6]. The separation of plasma oxylipins was conducted in a Shimadzu LC-10AD_VP_ instrument (Shimadzu Corp., Kyoto, Japan) equipped with a 2.1 mm X 150 mm **Pursuit XRs-C18** 5 μm column (Varian Inc, Palo Alto, CA) held at 40 ºC. A gradient of water containing 0.1% acetic acid (v/v, solvent A) and acetonitrile/methanol/acetic acid (800/150/1, v/v; solvent B) was used to elute the column with the flow rate of 0.4 mL min^-1^ (SI Table 1). The injection volume was 10 μL and the samples were kept at 10 ºC in the auto sampler. Analytes were detected on a 4000 QTRAP (Applied Biosystems, Foster City, CA) hybrid, triple-quadrupole, and linear ion trap mass spectrometer equipped with a Turbo V ion source and operated in negative MRM mode. The source was operated in negative electrospray mode and the QTRAP was set as follows: CUR= 20 psi, GS1=50 psi, GS2=30 psi, IS=-4500 V, CAD= HIGH, TEM=400°C, ihe=ON, DP= - 60 V. The collision energies used for CAD ranged from -18 to -38 eV.

**SUPPLEMENTARY FIGURES**

**Figure S1. a)** Whole heart images from control and DIC-treated mice. With treatment, **b**) there was a trend towards an increase in heart weight (HW), **c**) a reduction in body weight (BW), and **d**) an increase in HW to BW ratio. **e)** After 4 weeks, there was an increase in mortality in mice treated with DIC (p=0.06). **f)** Cardiac sections stained with Masson’s Trichrome from control and DIC-treated mice. Scale bar = 1 mm. Data expressed as mean ± SEM. Statistical significance was considered to be achieved when *P*<0.05 by *t*-test.

**Figure S2: a)** Oxylipin profile from arachidonic acid metabolites through the CYP450 pathway and **b)** 12/15 LOX pathway**.** Data expressed as mean ± SEM. n=9-16 mice for each group. Statistical significance was considered to be achieved when *P*<0.05 by *t-*test.

**Reference:**

1. Thai PN, Daugherty DJ, Frederich BJ, Lu X, Deng W, Bers DM, et al. Cardiac-specific Conditional Knockout of the 18-kDa Mitochondrial Translocator Protein Protects from Pressure Overload Induced Heart Failure. Scientific Reports. 2018;8(1):16213.

2. Sirish P, Ledford HA, Timofeyev V, Thai PN, Ren L, Kim HJ, et al. Action Potential Shortening and Impairment of Cardiac Function by Ablation of Slc26a6. Circ Arrhythm Electrophysiol. 2017;10(10):e005267.

3. Sirish P, Lopez JE, Li N, Wong A, Timofeyev V, Young JN, et al. MicroRNA profiling predicts a variance in the proliferative potential of cardiac progenitor cells derived from neonatal and adult murine hearts. J Mol Cell Cardiol. 2012;52(1):264-72.

4. Zhang XD, Timofeyev V, Li N, Myers RE, Zhang DM, Singapuri A, et al. Critical roles of a small conductance Ca²⁺-activated K⁺ channel (SK3) in the repolarization process of atrial myocytes. Cardiovasc Res. 2014;101(2):317-25.

5. Mitchell GF, Jeron A, Koren G. Measurement of heart rate and Q-T interval in the conscious mouse. Am J Physiol. 1998;274(3):H747-51.

6. Morisseau C, Goodrow MH, Newman JW, Wheelock CE, Dowdy DL, Hammock BD. Structural refinement of inhibitors of urea-based soluble epoxide hydrolases. Biochem Pharmacol. 2002;63(9):1599-608.
